# Overexpression of Tumor Necrosis Factor-Like Ligand 1 A in Myeloid Cells Aggravates Liver Fibrosis in Mice

**DOI:** 10.1155/2019/7657294

**Published:** 2019-02-14

**Authors:** Jinbo Guo, Yuxin Luo, Fengrong Yin, Xiaoxia Huo, Guochao Niu, Mei Song, Shuang Chen, Xiaolan Zhang

**Affiliations:** ^1^Department of Gastroenterology, The Second Hospital of Hebei Medical University, Hebei Key Laboratory of Gastroenterology, Hebei Institute of Gastroenterology, Shijiazhuang, Hebei, China; ^2^Department of Pediatric and Department of Biomedical Science, Cedars Sinai Medical Center, Los Angeles, USA

## Abstract

Macrophages are the master regulator of the dynamic fibrogenesis–fibrosis resolution paradigm. TNF-like ligand 1 aberrance (TL1A) was found to be able to induce intestinal inflammation and fibrosis. Furthermore, significantly increased TL1A had been detected in liver tissues and mononuclear cells of patients with primary biliary cirrhosis (PBC). This study was to investigate the effect of myeloid cells with constitutive TL1A expression on liver fibrogenesis. We found that TL1A expressions in liver tissues and macrophages were significantly increased in mice with liver fibrosis induced by injection of carbon tetrachloride (CCl_4_). TL1A overexpression in myeloid cells induced liver function injury, accelerated the necrosis and apoptosis of hepatocytes, recruited macrophages, and promoted activation of hepatic stellate cells (HSCs) and fibrosis. In vitro results of our study showed that TL1A overexpression in macrophages promoted secretion of platelet-derived growth factor-BB (PDGF-BB), tumor necrosis factor-*α* (TNF-*α*), and interleukin-1*β* (IL-1*β*). Culturing macrophages with TL1A overexpression could accelerate the activation and proliferation of primary HSCs. These results indicated that constitutive TL1A expression in myeloid cells exacerbated liver fibrosis, probably through macrophage recruitment and secretion of proinflammatory and profibrotic cytokines.

## 1. Introduction

Hepatic fibrosis is a common pathological consequence of chronic liver diseases, which is characterized by an extensive deposition of the extracellular matrix (ECM) mainly secreted by activated hepatic stellate cells (HSCs) [1]. HSC activation is the leading cause of liver fibrogenesis, and persistent chronic liver inflammation plays a vital role in HSC activation. It has been well known that macrophage plays an essential role during inflammatory, wound healing responses, as well as perpetuation of fibrosis [2]. In the stage of liver fibrogenesis, macrophages not only secrete proinflammatory and profibrotic cytokines which worsen hepatocellular damage and activate HSCs but also release chemokines such as C–C chemokine ligand 2 (CCL2) which recruit monocytes/macrophages in blood [3]. In recent years, the roles of macrophage in liver fibrosis have been emphasized. Chu et al. [4] found that C-C motif chemokine receptor 9-positive macrophages activated HSCs and promoted liver fibrosis, Lodder et al. [5] demonstrated that macrophage autophagy protected against liver fibrosis in mice, and He et al. [6] identified that myeloid-specific disruption of recombination signal-binding protein-Jkappa ameliorated hepatic fibrosis by attenuating inflammation.

Tumor necrosis factor-like ligand 1 A (TL1A), also known as vascular endothelial growth inhibitor (VEGI), is a recently recognized member of the TNF superfamily. TL1A is a transmembrane protein predominant in endothelial cells, playing a critical role in multiple chronic inflammatory processes [7]. It was illuminated that TL1A expressions were increased in serum, colonic tissues, and macrophages in mice with chronic colitis. Mice with constitutive TL1A expression in myeloid cells (Kupffer cells and macrophages) exhibited enhanced intestinal and colonic inflammation and fibrosis compared to wild-type (WT) littermates [8, 9]. Furthermore, TL1A antibody could alleviate the inflammatory response and fibrosis of the colon [10]. Other than colonic disease, the roles of TL1A in organ fibrosis were also found in rheumatoid arthritis (RA), myocardial fibrosis, and idiopathic pulmonary fibrosis [11–13]. However, few studies focused on the roles of TL1A in liver fibrogenesis. Recently, it was reported that TL1A expression was markedly increased in the patients with primary biliary cirrhosis (PBC) [14]. It was mainly located in the Kupffer cells (KCs) and infiltrated macrophages in the liver, indicating that TL1A might play an important role in liver fibrosis. Pradere et al. [15] demonstrated that there was no contribution of dendritic cells to liver fibrosis development. Therefore, our research focused on the effect of TL1A overexpression in myeloid cells on liver fibrogenesis.

## 2. Materials and Methods

### 2.1. Mice and Experimental Models

The animal experiments were carried out in strict accordance with the recommendations in the Guide for the Care and Use of Laboratory Animals by the National Institutes of Health. The Chinese Academy of Sciences Animal Care and Use Committee gave approval for the animal experiments. Age-matched wild-type (WT) C57BL/6 mice and transgenic mice (Tg) with TL1A overexpression in myeloid cells (macrophages, dendritic cells), aged 6 to 8 weeks and weighted 18-21 g, were used in experiments, provided by the Experimental Animal Center of the Second Affiliated Hospital of Hebei Medical University (qualification certificate no. 911102) and by Cedars-Sinai Medical Center (America), respectively. Mice were genotyped by PCR with tail DNA as a template. Repeated administration of CCl_4_ has become one of the most commonly used experimental models for inducing toxin-mediated liver fibrosis. CCl_4_ is widely used as a solvent for dissolving nonpolar compounds, such as fats and oils. Olive oil was used to dissolve CCl_4_ in our study. The WT mice and Tg mice were randomly divided into six groups (10 mice per group): Control/WT group, Oil/WT group, CCl_4_/WT group, Control/Tg group, Oil/Tg group, and CCl_4_/Tg group. The CCl_4_/WT and CCl_4_/Tg groups were injected intraperitoneally with 10% CCl_4_ (5 *μ*L/g, Sigma, St. Louis, MO) twice a week for 4 weeks to establish the hepatic fibrosis model. The Oil/WT and Oil/Tg groups were injected with olive oil, whereas the Control/WT group and Control/Tg group were injected with normal saline. All injections were given for 8 times. Mice were sacrificed at 48 h after the last injection. Blood was obtained from the retroorbital sinus of mice.

### 2.2. Detection of Liver Function

The levels of alanine aminotransferase (ALT), aspartate aminotransferase (AST), and total bilirubin (TBIL) in the serum were analyzed using an automatic biochemical analyzer (BECKMAN COULTER CX9, America).

### 2.3. Histological Analysis

Five *μ*m-thick paraffin-embedded liver sections were stained with hematoxylin and eosin (H&E) and Sirius Red. The necrosis area and positive area were calculated by the software of Image-Pro Plus. For immunohistochemical analysis, liver specimens were fixed in 10% buffered formalin and incubated with *α*-smooth muscle actin antibody (*α*-SMA, Sigma) and Collagen1*α*1 antibody (Affinity, USA), followed by the appropriate secondary antibodies. For dual-color immunofluorescence, sections were incubated with F4/80 antibody (Abcam, USA), TL1A antibody (Affinity, USA), and fluorescent secondary antibodies. The positive areas showed the color of brown yellow. Immunohistochemical analysis was performed with the software of Image-Pro Plus for calculating integrate optical density (IOD). Apoptosis was assessed by the terminal deoxynucleotidyl transferase dUTP nick end labeling (TUNEL) assay (Promega, Madison, WI, USA, #G7132). The number of TUNEL-positive nuclei was determined in 10 randomly selected fields. To detect hydroxyproline, 100 mg of wet liver samples was subjected to acid hydrolysis according to the protocol in the Hydroxyproline Testing Kit (Nanjing Jiancheng Bioengineering Co. Ltd., China).

### 2.4. Cell Isolation and Culture

Bone marrow cells were obtained by flushing the femur and tibia. The culture medium contained 10% fetal bovine serum (FBS) and 10 ng/mL macrophage colony-stimulating factor (M-CSF). After culture for 10 d, 100 ng/mL lipopolysaccharide (LPS) and 25 ng/mL interferon-*γ* (IFN-*γ*) were added into the culture medium to activate and differentiate the bone marrow cells into bone marrow-derived macrophages (BMMs). The medium of BMMs would be collected 3 d after adding LPS and IFN-*γ* for cytokine detection or as conditioned medium (CM) for culture of HSCs. Peritoneal macrophages (PMs) were obtained by flushing the enterocoelia and cultured with culture medium (containing 10% of FBS) for 6 to 8 h. After PMs became adhesive, 100 ng/mL LPS and 25 ng/mL IFN-*γ* were added into the culture medium. The medium of PMs would be collected 3 d after adding LPS and IFN-*γ* for cytokine detection or as CM for culture of HSCs.

HSCs were isolated as previously described [16]. Briefly, the liver was digested by pronase and collagenase, followed by density gradient centrifugation. More than 95% of the purity of isolated HSCs was confirmed by immunostaining with anti-Desmin antibody. To detect the effect of macrophages on the activation and proliferation of HSCs, HSCs were cultured with CM from BMMs/PMs for 24 h. The groups would be deducted or added in some experiments according to the different aims. We divided the Control group (HSCs from C57BL/6 mice with DMEM), M-CSF + LPS + IFN-*γ* group (HSCs from C57BL/6 mice with DMEM containing M-CSF, LPS, and IFN-*γ*), CM/WT group (HSCs from C57BL/6 mice incubated in medium containing 40% CM from M-CSF + LPS + IFN-*γ*-stimulated BMMs from WT mice for 24 h), and CM/Tg group (HSCs from C57BL/6 mice incubated in medium containing 40% CM from M-CSF + LPS + IFN-*γ*-stimulated BMMs from Tg mice for 24 h). PM-based cells were divided into the Control group (HSCs from C57BL/6 mice with DMEM), LPS + IFN-*γ* group (HSCs from C57BL/6 mice with DMEM containing LPS and IFN-*γ*), CM/WT group (HSCs from C57BL/6 mice incubated in medium containing 40% CM from LPS + IFN-*γ*-stimulated PMs from WT mice for 24 h), and CM/Tg group (HSCs from C57BL/6 mice incubated in medium containing 40% CM from LPS + IFN-*γ*-stimulated PMs from Tg mice for 24 h).

### 2.5. Cell Immunofluorescence and ELISA Detection

The expressions of TL1A in macrophages were detected by immunofluorescence staining as previously described [5]. The immunofluorescence staining of F4/80 was used for macrophage identification. To detect the effect of high expression of TL1A cells on the activation of HSCs, HSCs were cultured with 40% conditioned medium (CM) from BMMs/PM. The expressions of *α*-SMA in HSCs were detected by immunofluorescence on the 2nd, 4th, and 6th day, respectively. TGF-*β*1, PDGF-BB, TNF-*α*, and IL-1*β* in serum and CM were detected under the recommended protocols. ELISA kits were purchased from Lianke Biotech Co. Ltd.

### 2.6. In Vitro Primary HSC Proliferation Assay

Cell proliferation was assessed by the Cell Counting Kit-8 (CCK-8) assay (Dojindo, Japan) according to the manufacturer's protocol. Briefly, cells were seeded in 96-well plates (2 × 10^3^ cells/well) and incubated at 37°C with 5% CO_2_ for 24 h. Then cells were incubated with 50% conditional medium from macrophages and cultured for an additional 24 h. For CCK-8 detection, 10 *μ*L of CCK-8 solution was added into the wells and the cells were incubated at 37°C for 3 h. The absorbance (optical density (OD)) of cells was determined at 450 nm using a microplate reader.

### 2.7. Western Blot Analysis

Collagen 1*α*1, MMP-2, MMP-9, *α*-SMA, and CCL2 in liver tissues were detected. Preparation of protein extracts, electrophoresis, and subsequent blotting were performed as previously described [17]. The antibodies were all purchased from Sigma-Aldrich Co. LLC.

### 2.8. Real-Time Polymerase Chain Reaction (RT-PCR)

RNA was extracted from liver tissue and cultured cells using the RNeasy and DNase Kits (QIAGEN, Valencia, CA, USA) and was reverse transcribed using the High-Capacity cDNA Reverse Transcription Kit (Applied Biosystems, Foster City, CA, USA). Quantitative RT-PCR was performed in duplicate with the QuantiTect SYBR Green PCR kit (QIAGEN, Mainz, Germany) using the LightCycler apparatus. QuantiTect primer assays (QIAGEN) were used to amplify the following mRNAs: TL1A, Collagen1*α*1, *α*-SMA, MMP-2, MMP-9, TGF-*β*1, PDGF-BB, TNF-*α*, pro-IL-1*β*, F4/80, and CCL2 in liver tissues and *α*-SMA in HSCs. Oligonucleotide sequences used for RT-PCR analysis could be found in Table 1.

### 2.9. Statistical Analysis

Statistical analyses were performed using SPSS for Windows version 22.0 (SPSS Inc., Chicago, Illinois) and were expressed as mean ± standard deviation of the mean (SEM). The one-way ANOVA, the Student-Newman-Keuls (SNK) test, the Nemenyi test, and the Kruskal-Wallis test were used to assess the differences between groups, as appropriate. The level of statistical significance was set at *p* < 0.05 for all the tests.

## 3. Results

### 3.1. TL1A Was Upregulated in Liver Tissues and Macrophages in Mice with Hepatic Fibrosis

**Figure 1:**
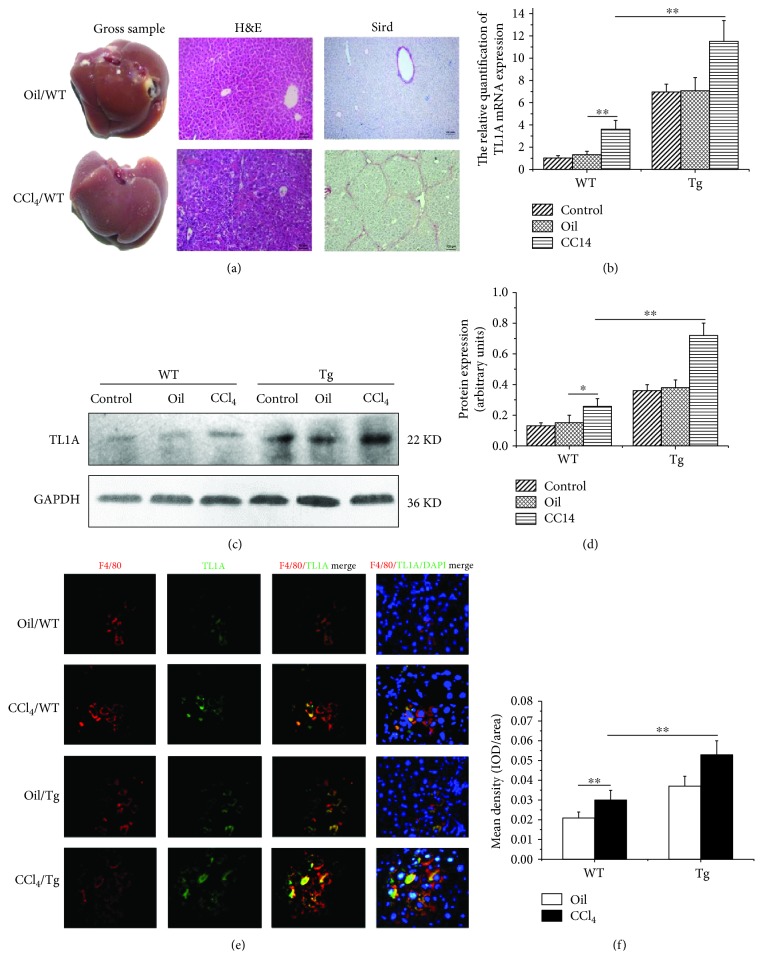
TL1A was upregulated in liver tissues and macrophages in mice with hepatic fibrosis. (a) The hepatic fibrosis model was established and verified by H&E and Sirius Red staining (200x). (b) The relative quantification of TL1A mRNA was measured by RT-PCR. (c, d) TL1A expression in the CCl_4_/WT group was significantly higher than those in the Control/WT and Oil/WT group, which was markedly higher in the CCl_4_/Tg group than that in the CCl_4_/WT group detected by Western blot. (e, f) TL1A was discovered in macrophage through F4/80 and TL1A immunofluorescence double staining, which was markedly higher in the CCl_4_/WT group than in the Oil/WT group and significantly higher in the CCl_4_/Tg group than in the CCl_4_/WT group. Data are expressed as mean ± SD, ^∗^*p* < 0.05 and ^∗∗^*p* < 0.01.

The hepatic fibrosis model was established successfully verified by H&E and Sirius Red staining (Figure 1(a)). The RT-PCR was performed to analyze TL1A mRNA expression levels in liver tissues. There was no significant difference in terms of TL1A mRNA expression between the Control/WT group and Oil/WT group (1 ± 0.02 vs 1.29 ± 0.31, *p* > 0.05), and the expression of TL1A mRNA in the CCl_4_/WT group was significantly higher than that in the Oil/WT group (3.57 ± 0.81 vs 11.5 ± 1.87, *p* < 0.01) (Figure 1(b)). As expected, TL1A expression in the CCl_4_/Tg group was markedly increased, as compared with that in the Oil/Tg group (11.5 ± 1.87 vs 7.08 ± 1.15, *p* < 0.01). Furthermore, the expression of TL1A protein was also shown with significantly higher levels in the CCl_4_/WT group (0.26 ± 0.05) and CCl_4_/Tg group (0.72 ± 0.08) compared with the equivalent Oil/WT group (0.15 ± 0.05) and Oil/Tg group (0.38 ± 0.05) detected by Western blot (all *p* < 0.01) (Figures 1(c) and 1(d)). F4/80 is the surface marker of macrophages. TL1A expression in macrophages was detected by dual-color immunofluorescence. As shown in Figures 1(e) and 1(f), the red and green fluorescence areas represented the expressions of F4/80 and TL1A, respectively. The mean integral optical density (IOD) of coexpression areas was evaluated. The IOD of the CCl_4_/WT group was obviously higher than that of the Oil/WT group (0.031 ± 0.005 vs 0.021 ± 0.003, *p* < 0.01). In addition, the IOD in the CCl_4_/Tg group was significantly higher than that in the CCl_4_/WT group (0.053 ± 0.007 vs 0.031 ± 0.005, *p* < 0.01). It was demonstrated that TL1A expression was increased in macrophages in liver tissue of mice with liver fibrosis.

### 3.2. High Expression of TL1A in Myeloid Cells Worsened Hepatic Inflammation

**Figure 2:**
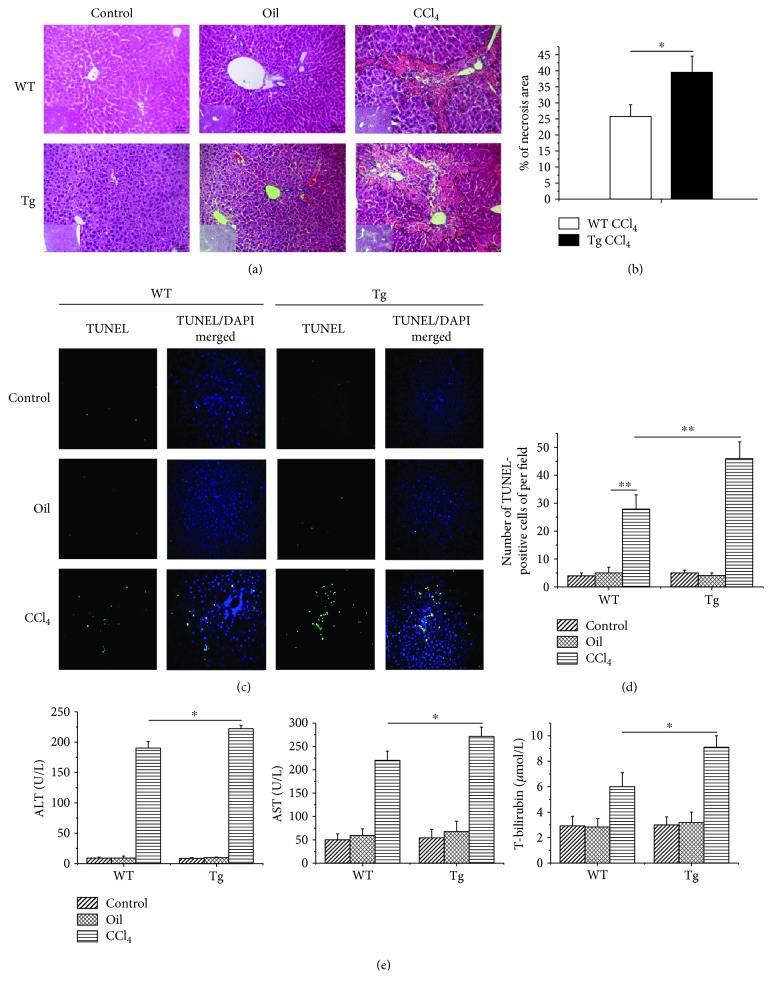
Constitutive TL1A expression on myeloid cells aggravated liver injury, hepatocyte necrosis, and apoptosis. (a, b) H&E staining showed that the hepatocyte necrosis area in the CCl_4_/Tg group was significantly larger than that in the CCl_4_/WT group (200x). (c, d) The apoptotic hepatocytes were significantly increased in the CCl_4_/WT group than in the Control/WT group and Oil/WT group, and more apoptotic hepatocytes were found in the CCl_4_/Tg group, compared with the CCl_4_/WT group (200x). (e) Serum ALT, AST, and TBIL in the CCl_4_/Tg group were significantly higher than those in the CCl_4_/WT group. Data are expressed as mean ± SD, ^∗^*p* < 0.05 and ^∗∗^*p* < 0.01.

We next evaluated the consequences of overexpression of TL1A in myeloid cells on chronic liver injury in mice exposed to repeated injections of CCl_4_ by studying the extent of hepatocyte necrosis and apoptosis. Analysis of liver histology in H&E staining showed that hepatocyte death was more pronounced in TL1A-Tg mice than in WT counterparts (Figures 2(a) and 2(b)). Moreover, the number of TUNEL-positive hepatocytes was significantly larger in CCl_4_-exposed TL1A-Tg mice than in WT animals (46 ± 6 vs 28 ± 5, *p* < 0.01) (Figures 2(c) and 2(d)). The liver function test also showed that serum ALT, AST, and TBIL levels were markedly elevated in TL1A-Tg mice exposed to CCl_4_ than in WT counterparts (ALT: 222.4 U/L ± 5.2 U/L vs 190.4 U/L ± 10.4 U/L, *p* < 0.05; AST: 70.8 U/L ± 19.7 U/L vs 220.5 U/L ± 19.3 U/L, *p* < 0.05; and TBIL: 9.09 *μ*mol/L ± 0.9 *μ*mol/L vs 6 *μ*mol/L ± 1.07 *μ*mol/L, *p* < 0.05) (Figure 2(e)).

### 3.3. Overexpression of TL1A in Myeloid Cells Aggravated Hepatic Fibrosis

**Figure 3:**
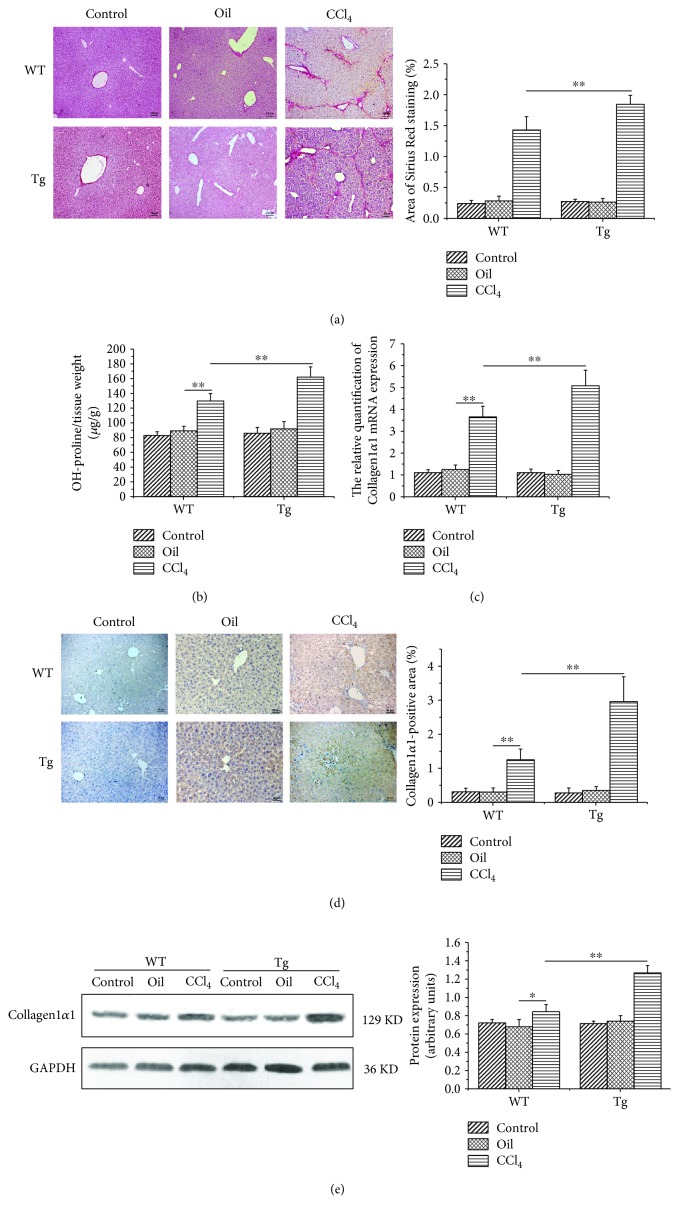
Constitutive TL1A expression on myeloid cells exacerbated liver fibrosis. (a) The Sirius Red-positive area in the CCl_4_/Tg group was markedly larger than that in the CCl_4_/WT group (200x). (b) The hydroxyproline content in the CCl_4_/Tg group was higher than that in the CCl_4_/WT group. (c–e) The Collagen1*α*1 expressions in the CCl_4_/Tg group were remarkably higher than those in the CCl_4_/WT group detected by RT-PCR, immunohistochemical staining (200x), and Western blot. Data are expressed as mean ± SD, ^∗^*p* < 0.05 and ^∗∗^*p* < 0.01.

We evaluated hepatic fibrosis by Sirius Red staining and hydroxyproline determination. The positive area of fibrous connective tissue showed obviously higher levels in TL1A-Tg mice compared with WT mice exposed to repeated injections of CCl_4_ (1.85%±0.14% vs 1.43%±0.21%, *p* < 0.05) (Figure 3(a)). Hydroxyproline content was also markedly higher in TL1A-Tg mice than in WT counterparts following CCl_4_ exposure (162 *μ*g/g ± 14 *μ*g/g vs 130 *μ*g/g ± 10 *μ*g/g, *p* < 0.05) (Figure 3(b)). Collagen1*α*1 is the major component of total collagen constituting the ECM, which is also used for measuring the degree of fibrosis. Collagen1*α*1 detected by RT-PCR, Western blotting, and immunohistochemical analysis showed similar tendencies with significantly higher levels in the CCl_4_/Tg group compared with the CCl_4_/WT group (Figures 3(c)–3(e)).

### 3.4. Overexpression of TL1A in Myeloid Cells Promoted the Activation of HSCs

**Figure 4:**
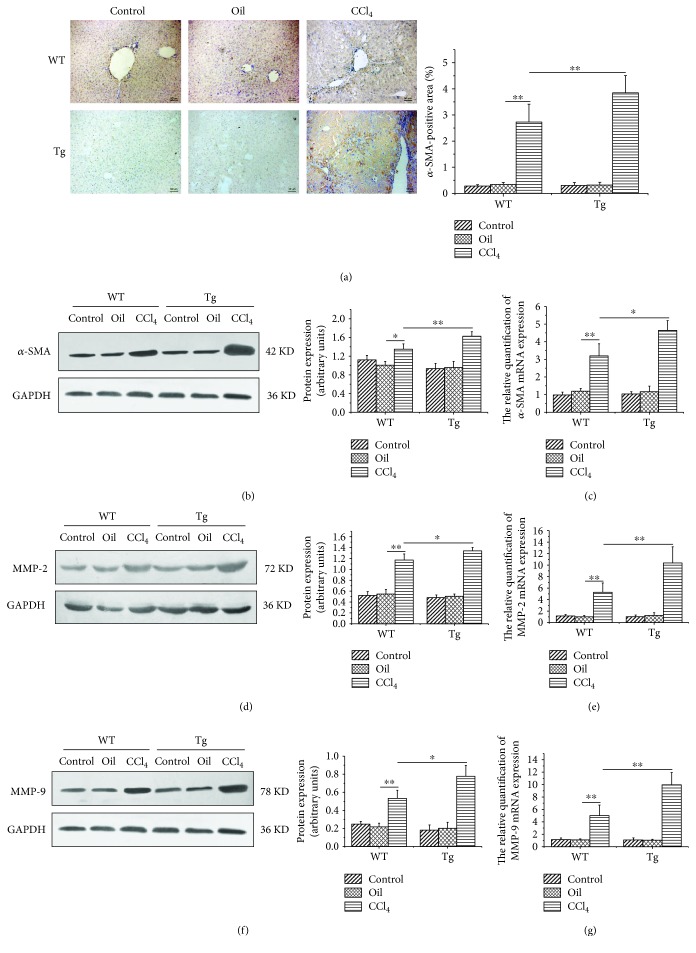
Constitutive TL1A expression on myeloid cells facilitated HSC activation and the expressions of MMP-2 and MMP-9. (a–c) The *α*-SMA expressions in the CCl_4_/Tg group were markedly higher than that in the CCl_4_/WT group detected by immunohistochemical staining (200x), Western blot, and RT-PCR. (d, e) The MMP-2 expressions in the CCl_4_/Tg group were significantly increased than those in the CCl_4_/WT group detected by Western blot and RT-PCR. (f, g) The MMP-9 expressions in the CCl_4_/Tg group were notably increased than those in the CCl_4_/WT group detected by Western blot and RT-PCR. Data are expressed as mean ± SD, ^∗^*p* < 0.05 and ^∗∗^*p* < 0.01.

The activation of HSCs is pivotal in hepatic fibrosis [1]. The *α*-SMA, as the marker of HSC activation, was detected. Immunostaining of liver sections showed that the levels of *α*-SMA increased remarkably in the livers of TL1A-Tg mice as compared with the Control after CCl_4_ injection (Figure 4(a)), indicating that TL1A overexpression activated HSCs more extensively. The Western blotting and RT-PCR results of *α*-SMA also showed significantly higher levels in the CCl_4_/Tg group compared with that in the CCl_4_/WT group (Figures 4(b) and 4(c)). Gelatinases, including MMP-2 and MMP-9, were involved in the degradation of ECM components, which promoted the activation and proliferation of HSCs by degrading the basilar membrane. The expressions of MMP-2 and MMP-9 were detected by RT-PCR and Western blotting. The levels of MMP-2 protein and mRNA were significantly higher in the CCl_4_/Tg group than in the CCl_4_/WT group (Figures 4(d) and 4(e)). Similar tendency was observed in MMP-9 as in MMP-2 (Figures 4(f) and 4(g)).

### 3.5. Overexpression of TL1A in Myeloid Cells Enhanced Macrophage Recruitment

**Figure 5:**
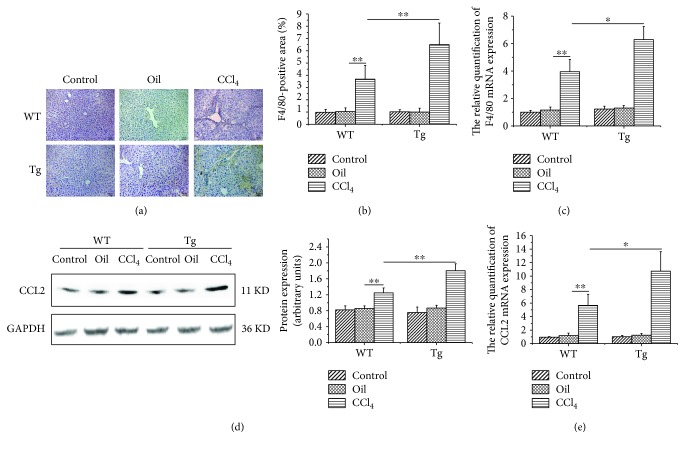
Constitutive TL1A expression on myeloid cells promoted macrophage recruitment to the liver. (a–c) The expression of F4/80 was significantly higher than that in the CCl_4_/WT group detected by immunohistochemical staining (200x) and RT-PCR. (d, e) CCL2 in the CCl_4_/Tg group was significantly higher than that in the CCl_4_/WT group detected by Western blot and RT-PCR. Data are expressed as mean ± SD, ^∗^*p* < 0.05 and ^∗∗^*p* < 0.01.

Several studies have emphasized the crucial role of infiltrating macrophages for the progression of liver inflammation and fibrosis in experimental mouse models [2, 4, 6]. Immunohistochemical staining and RT-PCR were used to detect F4/80 expression, which reflected macrophage infiltration. TL1A-Tg mice showed significantly increased liver macrophages after CCl_4_-induced liver damage (Figures 5(a)–5(c)). Under conditions of liver damage, CCL2 promoted monocyte subset infiltration into the liver. We found markedly elevated CCL2 protein and mRNA in the CCl_4_/Tg group compared with the CCl_4_/WT group (Figures 5(d) and 5(e)).

### 3.6. Overexpression of TL1A in Myeloid Cells Increased the Expressions of PDGF-BB, TNF-*α*, and IL-1*β* in Serum, Liver Tissue, and Conditioned Medium for Macrophages

**Figure 6:**
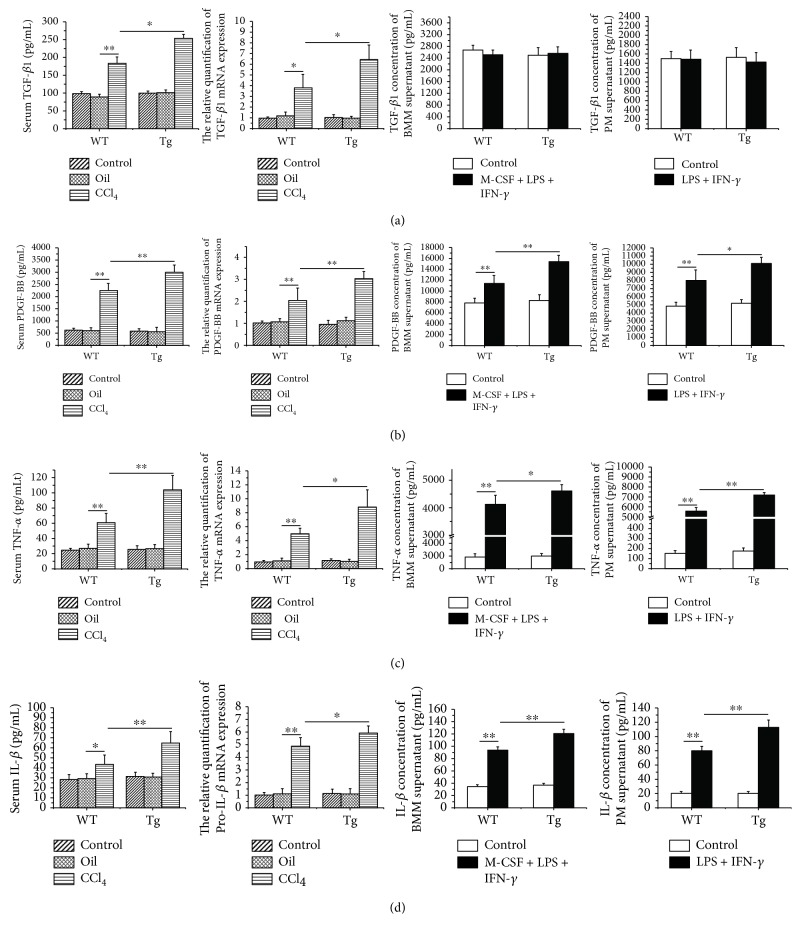
Constitutive TL1A expression on macrophages upregulated the PDGF-BB, TNF-*α*, and IL-1*β* in the serum, liver tissues, and macrophages. (a) The expression of TGF-*β*1 in serum, liver tissue, and the medium of BMMs and PMs was detected by ELISA and RT-PCR. Serum TGF-*β*1 in the CCl_4_/Tg group were significantly higher than those in the CCl_4_/WT group, and TGF-*β*1 mRNA in the CCl_4_/Tg group were also higher than those in the CCl_4_/WT group. But there was no statistical significance between the Tg group and WT group of CM derived from BMMs and PMs. (b, c, d) The expressions of PDGF-BB, TNF-*α*, and IL-1*β* in serum, liver tissue, and the medium of BMMs and PMs were detected by ELISA and RT-PCR, respectively. The cytokines in serum and mRNA in liver tissue in the CCl_4_/Tg group were significantly higher than those in the CCl_4_/WT group. And those derived from BMM and PM supernatant in the LPS + IFN-*γ* + M-CSF/Tg group were significantly higher than those in the LPS + IFN-*γ* + M-CSF/WT group. Data are expressed as mean ± SD, ^∗^*p* < 0.05 and ^∗∗^*p* < 0.01.

TGF-*β*1 is considered to accelerate liver fibrosis mainly secreted by activated HSCs, stimulating collagen gene transcription and inhibiting degradation of ECM [18]. As shown in Figure 6(a), TGF-*β*1 mRNA in liver tissue by RT-PCR was shown with a significantly higher level in the CCl_4_/Tg group compared with the CCl_4_/WT group (6.45 ± 1.35 vs 3.83 ± 1.23, *p* < 0.05) and the TGF-*β*1 level in serum by ELISA showed the similar tendency as well (253.6 pg/mL ± 11.4 pg/mL vs 184 pg/mL ± 17.3 pg/mL, *p* < 0.05). However, TGF-*β*1 concentration between CM for macrophages derived from TL1A-Tg mice and WT mice was not significantly different. PDGF-BB is essential for HSC proliferation which was secreted by macrophages, blood platelets, and activated HSCs. Remarkable elevations of PDGF-BB mRNA in liver tissue and PDGF-BB level in serum were observed in the CCl_4_/Tg group compared with the CCl_4_/WT group. Moreover, PDGF-BB concentration in CM for BMMs and PMs derived from TL1A-Tg mice was markedly higher than that from WT mice (Figure 6(b)). TNF-*α* and IL-1*β* are mostly produced by macrophages and infiltrated monocytes and involved in chronic hepatic inflammation and fibrosis. The results showed that TNF-*α* and IL-1*β* were remarkably higher in serum, liver tissue, and CM obtained from TL1A-Tg mice than WT mice (Figures 6(c) and 6(d)).

### 3.7. Overexpression of TL1A in Macrophages Promoted Activation and Proliferation of HSCs

**Figure 7:**
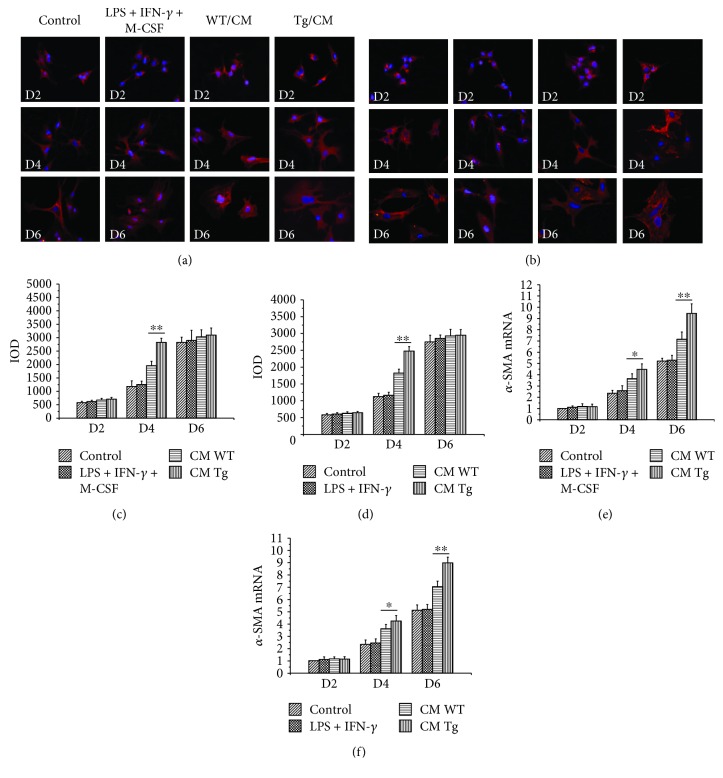
Constitutive TL1A expression on macrophages promoted the activation of HSCs. (a, c, e) The *α*-SMA expressions of HSCs cultured with BMM-derived CM in the CM/Tg group were markedly higher than those in the CM/WT group detected by immunofluorescence staining (400x) and RT-PCR. (b, d, f) The *α*-SMA expressions of HSCs cultured with PM-derived CM in the CM/Tg group were significantly higher than those in the CM/WT group detected by immunofluorescence staining (400x) and RT-PCR. Data are expressed as mean ± SD, ^∗^*p* < 0.05 and ^∗∗^*p* < 0.01.

We further evaluated the impact of TL1A overexpression of macrophage on the activation and proliferation of HSCs in conditioned medium experiments. The *α*-SMA as the marker of HSC activation was detected by immunofluorescence and RT-PCR. The protein expression of *α*-SMA in HSCs cultured with CM of BMMs isolated from TL1A-Tg mice showed no significant difference compared to that derived from WT mice on the 2nd and 6th day. However, the protein expression of *α*-SMA was significantly increased in HSCs cultured with CM of BMMs with TL1A overexpression on the 4th day (Figures 7(a) and 7(c)), which was consistent with the results of PMs (Figures 7(b) and 7(d)). The mRNA expression of *α*-SMA was significantly enhanced in HSCs exposed to CM collected from macrophages (BMMs and PMs, respectively) with TL1A overexpression on the 4th and 6th day (Figures 7(e) and 7(f)). The proliferation rate was detected by CCK-8 assay. As shown in Table 2, the proliferation rate of HSCs exposed to CM collected from macrophages (BMMs and PMs, respectively) with TL1A overexpression was significantly higher than that of HSCs exposed to CM collected from macrophages isolated from WT mice.

## 4. Discussion

The onset and progression of fibrosis is closely linked to chronic inflammatory reactions. Recruitment of immune cells such as macrophages and T-cells to the site of injury is an important event in the initiation of inflammation, as well as for wound healing and hepatic fibrosis [19]. TL1A is a tumor necrosis factor family member expressed by monocytes, macrophages, dendritic cells (DCs), and endothelial cells in response to stimulation by cytokines, immune complexes [20]. TL1A exerts pleiotropic effects on cell proliferation, activation, and differentiation of immune cells, which involves autoimmune diseases such as IBD, RA, and ankylosing spondylitis (AS). It has been proved that serum TL1A levels were significantly increased in patients with PBC, chronic hepatitis C, or autoimmune hepatitis. And its levels were significantly decreased in early-stage PBC patients after ursodeoxycholic acid (UDCA) treatment. TL1A was immunohistochemically located to biliary epithelial cells, Kupffer cells, blood vessels, and infiltrating mononuclear cells in the PBC liver [14]. However, the role of TL1A in liver fibrosis remains poorly understood.

To investigate the roles of TL1A in the process of hepatic fibrosis, the CCl_4_-induced hepatic fibrosis model was established and a series of experiments at cellular, molecular, and protein levels were carried out in this study. Consistent with a previous clinical study that TL1A expression was found to be increased in serum and liver tissues in patients with PBC and advanced hepatitis cirrhosis [14], the present study showed that the expressions of TL1A protein and mRNA were significantly higher in mice with liver fibrosis than in the Control mice. In the clinical study on TL1A, mononuclear phagocytes are thought to be the main source of TL1A in patients with RA [21]. In the liver of PBC patients, TL1A expression is positive for infiltrating mononuclear cells as well. Therefore, we detected the TL1A expression in macrophages of liver tissues by dual-color immunofluorescence. As expected, TL1A was notably increased in macrophages after CCl_4_-induced liver damage.

The key role of macrophages in promoting hepatic fibrogenesis has been demonstrated in a number of studies. In fibrotic mouse livers, the accumulation of macrophages greatly augments the local macrophage pool, perpetuates inflammation causing further hepatocyte injury, and activates fibrogenic HSCs [22, 23]. Furthermore, in CD11b-DTR mice, selective depletion of macrophages during ongoing injury led to a reduction of liver fibrosis [24]. Shih et al. [9] showed that an increased number of macrophages were found in transgenic mice with overexpression of TL1A in an intestinal fibrosis model. Similarly, we also found that the macrophages in hepatic tissues were significantly increased in TL1A-Tg mice than in WT mice, confirming that the high expression of TL1A in myeloid cells during hepatic fibrosis promoted the recruitment of macrophages to the liver. In the chronically inflamed liver, CCL2 is secreted mainly by injured hepatocytes, biliary epithelial cells, and HSCs [25], which is associated with the infiltration of macrophages [26]. In our study, CCL2 expression was significantly higher in TL1A-Tg mice than in the WT mice, indicating that the promotion of TL1A in the recruitment of macrophages might be related to the upregulation of CCL2 expression in liver tissues.

Chronic persistent inflammation is a main cause of liver fibrosis. Liver inflammation induces necrosis and apoptosis in liver cells and releases enzymes in liver cells. In this study, the levels of ALT, AST, and TBIL in serum were significantly higher in TL1A-Tg mice than in the WT mice with liver fibrosis, indicating that overexpression of TL1A in myeloid cells aggravated hepatocyte injury. Moreover, the H&E staining and TUNEL assay revealed that the necrotic area of hepatocyte was significantly wider and the apoptosis number of hepatocytes was significantly larger in TL1A-Tg mice than in WT counterparts. The above results confirmed that overexpression of TL1A in myeloid cells caused more necrosis and apoptosis of liver cells, as well as more severe liver injury and inflammation.

HSCs are typically found in the space of Disse in a quiescent state, which are activated to myofibroblasts expressing *α*-SMA by several stimuli such as cytokines and inflammatory mediators. Collagen1*α*1, a major component of total collagen constituting the ECM, are mainly released by activated HSCs. The results revealed that *α*-SMA and Collagen1*α*1 expressions were markedly higher in TL1A-Tg mice than in WT mice detected by immunohistochemical staining, RT-PCR, and Western blot assay. Hydroxyproline is a special amino acid of collagen, and its content can reflect the total collagen condition and assess the degree of fibrosis. In this study, we found that the content of hydroxyproline in TL1A-Tg mice treated with CCl4 was significantly higher than that in WT mice. It was also confirmed that the TL1A-Tg mice were combined with more severe liver fibrosis by Sirius Red staining. In the early stage of hepatic fibrogenesis, gelatinases, including MMP-2 and MMP-9, are involved in the degradation of ECM components around HSCs. Previous studies have demonstrated that MMP-2 and MMP-9 promote the activation and proliferation of HSCs by degrading the basilar membrane and increase the biological activity of IL-1*β* and TGF-*β* [27, 28]. Infiltrated macrophages are the important source of MMP-2 and MMP-9 [29]. Our study confirmed significantly higher levels of MMP-2 and MMP-9 in TL1A-Tg mice than in WT counterparts. The above results demonstrated that overexpression of TL1A in myeloid cells accelerated HSC activation, which might be related to macrophage recruitment, thereby releasing more MMP-2 and MMP-9.

The important mechanism for macrophages to participate in liver fibrosis is to secrete a series of proinflammatory and profibrotic factors, which can act on the HSCs to induce a profibrotic phenotype. Specifically, macrophages can produce and activate the archetypal profibrotic cytokine TGF-*β*1, which acts to increase myofibroblast ECM and TIMP-1 production [30]. Additionally, hepatic macrophages can produce PDGF-BB (a potent stimulator of myofibroblast proliferation), IL-1*β*, and TNF-*α* (proinflammatory cytokines) to perpetuate the proinflammatory pro-fibrotic stimulus [31]. Our study showed that overexpression of TL1A in myeloid cells promoted the expression of TGF-*β*1 in liver tissues and serum, whereas TL1A had no effect on the secretion of TGF-*β*1 by macrophages themselves. We speculated that the TGF-*β*1 was secreted primarily by M2 macrophages, which in our study involved M1 macrophages. The others including PDGF-BB, TNF-*α*, and IL-1*β* were obviously increased in serum and liver tissues in TL1A-Tg mice compared to WT mice. Consistent with in vivo experiment, the concentration of PDGF-BB, TNF-*α*, and IL-1*β* in CM of macrophages derived from TL1A-Tg mice was markedly higher than that from equivalent WT mice. The results in this study confirmed that TL1A was able to enhance the proinflammatory factor (PDGF-BB, TNF-*α*, and IL-1*β*) secretion of macrophages directly. We cultured primary HSCs with CM of macrophages with or without TL1A overexpression, respectively, to further investigate the activation and proliferation of HSCs. Previous studies had confirmed cultured HSCs with macrophage medium promoting the activation and proliferation of HSCs [5, 15, 32], which accorded with the present results. Additionally, macrophages with overexpression of TL1A accelerated the activation and proliferation more obviously.

In conclusion, overexpression of TL1A in myeloid cells could accelerate the process of hepatic fibrosis, worsen the liver function, promote macrophages recruitment, upregulate the levels of PDGF-BB, TNF-*α*, and IL-1*β* in liver tissues and macrophages, and further stimulate the activation and proliferation of HSCs.

## Figures and Tables

**Table 1:** Oligonucleotide sequences used for Q-PCR analysis.

Transcript		Sequence (5′-3′ direction)	Product size
TL1A	F R	CGGGGAGACGACCAAACAAG AAGGAGAACGTGGCCCCAAGGTAG	160 bp
*α*-SMA	F R	TGCTGTCCCTCTATGCCTCT CGGCCAGCCAAGTCCAGACG	133 bp
Collagen1*α*1	F R	ACGGGAGGGCGAGTGCTGTG GGGGCCAGGCACGGAAACTC	277 bp
MMP-2	F R	TTTCCTGGGCAACAAGTATGAGAGC CTGGTCAGTGGCTTGGGGTATCCTC	618 bp
MMP-9	F R	CACCGCCAACTATGACCAGGAT GTTTAGAGCCACGACCATACAG	398 bp
F4/80	F R	CCTGCCACAACACTCTCGGAAGC TGGGCATGAGCAGCTGTAGGATC	191 bp
TNF-*α*	F R	CCGCGACGTGGAACTGGCAGAAG CCGATCACCCCGAAGTTCAGTAG	150 bp
Pro-IL-1*β*	F R	ACAGATGAAGTGCTCCTTCCA GTCGGAGATTCGTAGCTGGAT	73 bp
PDGF-BB	F R	GGGGCTTCCAGGAGTGATACCA GCCCGAGCAGGTCAGAACAAA	163 bp
TGF-*β*1	F R	CGCCAGTCCCCCAAGCCA CGCGGGTGACCTCTTTAGCATAG	155 bp
CCL2	F R	GCTGGAGAGCTACAAGAGGATCA TCTCTCTTGAGCTTGGTGACAAAA	78 bp
GAPDH	F R	TCGTCCCGTAGACAAAATGG TTGAGGTCAATGAAGGGGTC	132 bp

**Table 2:** Effect of macrophages with high expression of TL1A on the proliferation of HSCs.

Cells	Groups			
	Control	LPS + IFN-*γ* + M-CSF	CM/WT	CM/Tg
BMMs	100.82 ± 8.48	100.54 ± 8.11	203.24 ± 10.50	246.21±8.34^∗∗^
PMs	100.00 ± 8.13	101.63 ± 7.97	162.23 ± 9.40	194.29±6.82^∗∗^

^∗∗^ *p* < 0.01 vs the CM/WT group.

## Data Availability

The data used to support the findings of this study are available from the corresponding author upon request.
